# A weakly supervised deep learning-based method for glioma subtype classification using WSI and mpMRIs

**DOI:** 10.1038/s41598-022-09985-1

**Published:** 2022-04-12

**Authors:** Wei-Wen Hsu, Jing-Ming Guo, Linmin Pei, Ling-An Chiang, Yao-Feng Li, Jui-Chien Hsiao, Rivka Colen, Peizhong Liu

**Affiliations:** 1 Department of Electrical Engineering, National Taiwan University of Science and Technology, Taipei, Taiwan, ROC; 2 Imaging and Visualization Group, ABCS, Frederick National Laboratory for Cancer Research, Frederick, MD 21702 USA; 3 Department of Pathology, Tri-Service General Hospital and National Defense Medical Center, Taipei, 11490 Taiwan, ROC; 4 Department of Radiology, University of Pittsburgh, Pittsburgh, PA 15232 USA; 5 Hillman Cancer Center, University of Pittsburgh Medical Center, Pittsburgh, PA 15260 USA; 6 College of Engineering, Huaqiao University, Quanzhou, China

**Keywords:** Cancer imaging, Machine learning, Computational models

## Abstract

Accurate glioma subtype classification is critical for the treatment management of patients with brain tumors. Developing an automatically computer-aided algorithm for glioma subtype classification is challenging due to many factors. One of the difficulties is the label constraint. Specifically, each case is simply labeled the glioma subtype without precise annotations of lesion regions information. In this paper, we propose a novel hybrid fully convolutional neural network (CNN)-based method for glioma subtype classification using both whole slide imaging (WSI) and multiparametric magnetic resonance imagings (mpMRIs). It is comprised of two methods: a WSI-based method and a mpMRIs-based method. For the WSI-based method, we categorize the glioma subtype using a 2D CNN on WSIs. To overcome the label constraint issue, we extract the truly representative patches for the glioma subtype classification in a weakly supervised fashion. For the mpMRIs-based method, we develop a 3D CNN-based method by analyzing the mpMRIs. The mpMRIs-based method consists of brain tumor segmentation and classification. Finally, to enhance the robustness of the predictions, we fuse the WSI-based and mpMRIs-based results guided by a confidence index. The experimental results on the validation dataset in the competition of CPM-RadPath 2020 show the comprehensive judgments from both two modalities can achieve better performance than the ones by solely using WSI or mpMRIs. Furthermore, our result using the proposed method ranks the third place in the CPM-RadPath 2020 in the testing phase. The proposed method demonstrates a competitive performance, which is creditable to the success of weakly supervised approach and the strategy of label agreement from multi-modality data.

## Introduction

Brain tumors, originating in the glial cells, are cancerous masses in the central nervous system (CNS)^1^. In 2011–2015, there are 23 out of 100,000 population diagnosed with brain tumors in the US^2^. Prior to 2016, World Health Organization (WHO) categorizes the CNS gliomas into grades I–IV based on the histological features of a heterogeneous population of the tumor^3^. However, a new brain tumor classification criterion was released from the WHO in 2016. According to the new criterion, the tumor classification is determined based on both phenotypic and genotypic information^4^. There are many glioma subtypes: diffuse astrocytoma, isocitrate dehydrogenase (*IDH)*-mutant/-wildtype, anaplastic astrocytoma *IDH*-mutant/-wildtype, oligodendroglioma, *IDH*-mutant and *1p/19q-codeleted*, glioblastoma, *IDH*-mutant/-wildtype, etc. The prognosis of a patient with brain tumors is highly related to the tumor grade^2^. In general, patients with higher-grade gliomas have a less survival period. Especially, for patients with glioblastoma (GBM), the median survival period still remains 12–16 months, even with treatment advancement^5^. Consequently, an accurate and robust glioma subtype prediction provides a valuable guide for diagnosis and treatment management. Conventionally, brain tumor diagnosis or grading is performed by pathologists, who examine tissue sections fixed on glass slides under a light microscope. Yet, the manual diagnosis/grading process is time-consuming and susceptible to human errors. Therefore, computer-aided brain tumor subtype classification is highly desired.

Prior to 2016, digital pathology images are the primary sources for the glioma subtype classification. Digital pathology is the digitized process of whole slide images (WSI) transforming into high-resolution images^6^. Digital pathology has become increasingly common because of the rich context information on the WSI. There are extensive studies on tumor subtype classification in the literature: Kothari et al*.* utilize a multi-class model for histological classification^7^. Chang et al*.* use the spatial pyramid matching framework (SPM) with a linear Support Vector Machine (SVM) classifier to classify glioblastoma multiforme (GBM)^8^. A hybrid machine learning method using SVM, random forest (RF), and neural network (NN) is proposed for glioma grading based on the WSI^9^. Barker et al*.* exploit an elastic net for brain tumor type classification^10^. However, a common limitation of these conventional machine learning methods is feature extraction, which requires professional clinical background and computer vision knowledge. In recent years, deep learning (DL)-based approaches have shown superior performance, and have been widely applied in many domains, e.g., computer vision^11^, medical image analysis^12,13^, and natural language processing (NLP)^14^. The deep learning-based methods is also adopted for glioma classification based on WSI^15^ and for glioma grading^16,17^.

On the other hand, MRI is an alternative source for glioma grading because of the noninvasive property. The MRI-based approaches also provide promising results for glioma classification and grading. Zacharaki et al*.* apply a SVM-based method to classify tumor type on MRI^18^. In the paper, they first extract radiological features, e.g., tumor shape and intensity characteristics. They then apply feature selection using a SVM with recursive feature elimination. Finally, they perform the tumor classification using another SVM. In addition, a hybrid method using a SVM and k-nearest neighbour classifiers (named as SVM-KNN) is also utilized for brain cancer classification^19^. In Refs.^20,21^, Random Forest (RF)-based methods are used for tumor classification as well. Recently, CNN-based methods have been becoming prevalent for such tasks^22,23^. Sajjad et al*.* propose a deep learning-based method for multi-grade brain tumor classification^24^. Liu et al*.* present a multi-task CNN algorithm for joint segmentation and genotype prediction of brainstem gliomas^25^. Pei et al*.* utilize a 3D CNN-based method for brain tumor subtype classification, and achieve the state-of-the-art performance^26^.

Unsurprisingly, a combination of pathology and radiology images provides more comprehensive context information than using a single modality alone. Ma et al*.* propose CNN-based methods for tumor classification on WSI and MRI^27–30^. Kurc et al*.* investigate brain tumor classification using machine learning and deep learning on WSI and MRI^31^. The work of using both WSI and MRI in Refs.^27–30^ offers state-of-the-art performance. However, all the methods are fusion-based at the feature level. We argue that these methods undermine the priority of pathology in tumor classification, which conflicts with the criterion defined by the WHO. We should develop a computer-aided diagnosis system and should take pathological modality as the primary source for tumor classification.

Therefore, we propose a novel hybrid fully convolutional neural network (CNN)-based method for glioma subtype classification using both whole slide image (WSI) and multiparametric magnetic resonance image (mpMRIs). The proposed method primarily focuses on the WSI-based result while taking the mpMRIs-based result as the complementary reference to enhance the robustness.

## Methodology

In this section, the proposed two approaches (the WSI-based approach and the mpMRIs-based approach) are elaborated. In addition, the label agreement strategy for fusing the prediction is also covered.

### Overall pipeline

**Figure 1:**
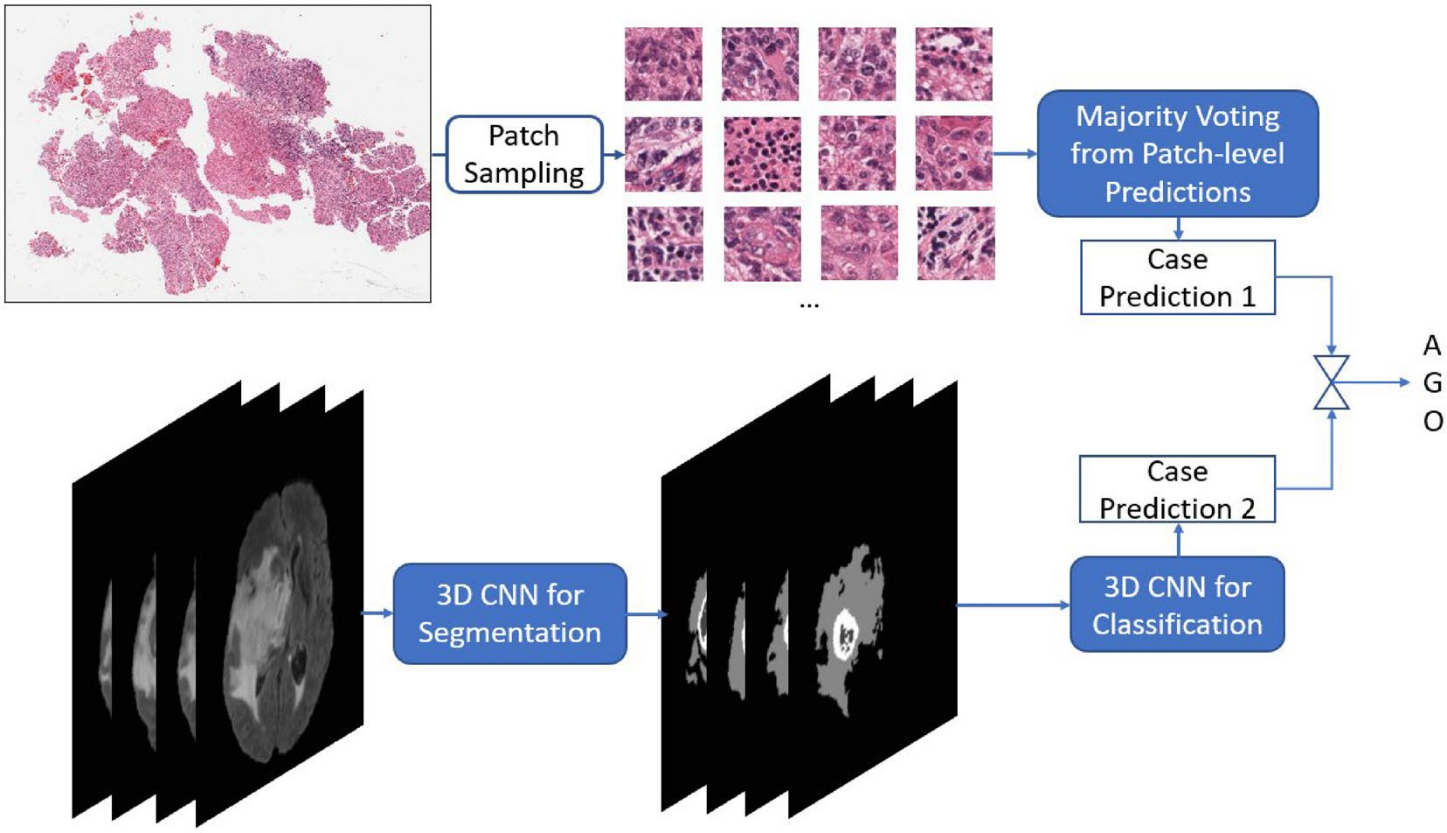
Overall pipeline of the proposed method in inference phase.

Figure 1 shows the overall pipeline of the proposed method. It consists of a WSI-based approach at the top and a mpMRIs-based approach at the bottom. Each approach outputs a probability of each subtype for each case. The final prediction is primarily derived from the WSI-based result. However, the final prediction is corrected as the mpMRIs-based result when the confidence index of WSI-based result is less than a threshold value. The threshold value is obtained in the validation phase of the Challenge, which achieves the best performance by using the proposed method.

### WSI-based approach

**Figure 2:**
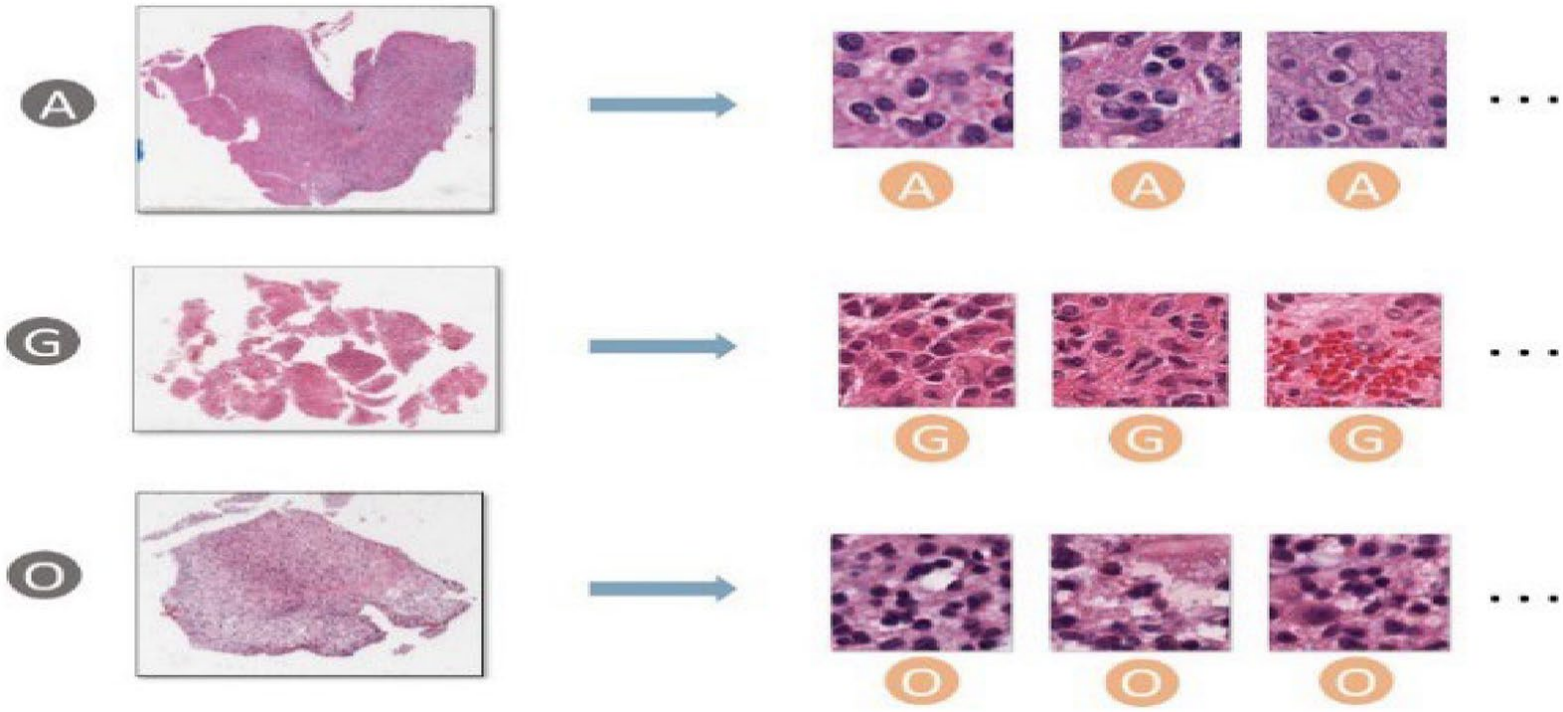
Patch sampling: Patches are randomly sampled from the densely cell-distributed areas of a WSI. All sampled patches have the same category as the label of the corresponding WSI.

**Figure 3:**
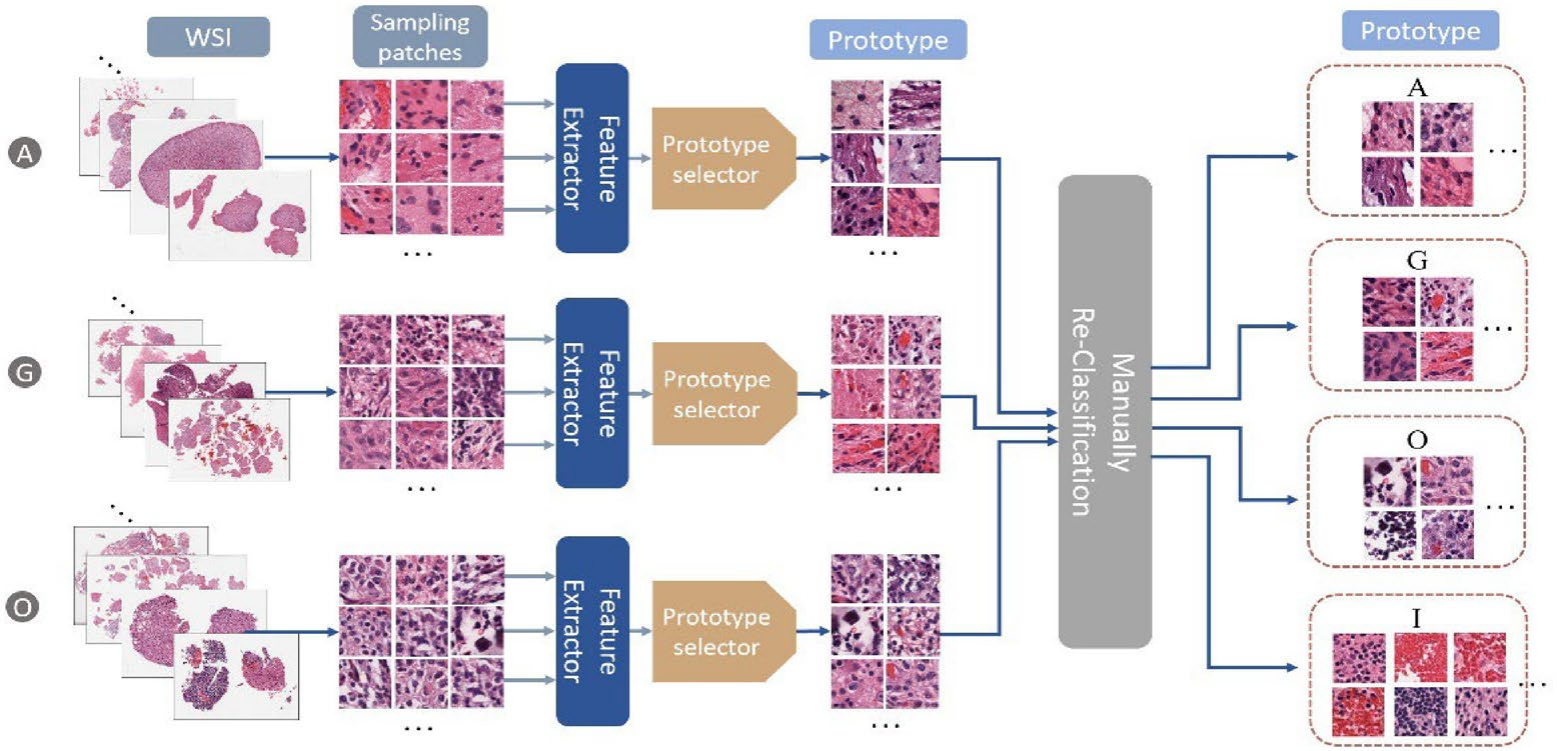
The pipeline of training phase in the WSI-based approach.

Even the training data with paired image and classification label is available in the CPM-RadPath 2020 challenge^32^, the classification task is still challenging because of the small number of cases and the label constrain issue. The label of the WSI is given, however, the precise lesion region information is missing. The issue becomes worse when considering the massive size of WSI. The inexact labeling in a weakly supervised learning task results in inaccurate samples in the training process. Extracting representative patches is of importance for the task. To overcome the label constrain issue, we extract multiple patches according to the intensity distribution, and assign the corresponding label, as shown in Fig. 2. These patch candidates are randomly selected from the areas with tissues on each WSI. In addition, to further screen out those samples without dense cell distribution, we apply two following criteria to the selecting patches: (1) The mean of all pixel intensities for each sampling patch should lie in between 50 and 150, and the standard deviation of pixel intensities in each channel of R, G, and B should be greater than 20. (2) The difference of maxima and minima of pixel intensity mean should be smaller than 100. The first condition ensures the selecting patches having rich context, rather than blank samples. The second condition screens out those patches that contain color markers. By following the rules, we extract 300 patches for each WSI in both the training and inference phases. Since the ratio of noisy samples is unknown and unpredictable, the prototype selection method can reduce the impact of noisy samples in the training process. Figure 3 shows the pipeline of the training phase in the WSI-based approach. First, all sampling patches for each category are collected to train using a ResNet50^33^. Subsequently, the trained CNN model is used to extract the convolutional representations (deep features) for each sampling patch. To reduce the impact of non-representative samples in each class, several prototypes for each category are selected by pair-wise similarity check. The extracted prototypes (e.g. 100 prototypes for each category in our experiment) are selected according to the similarity of intensity distribution. However, non-representative prototypes may also be collected because of the existence of irrelevant tissues such as lymphocytes, red blood cells, and mostly stroma, etc. In addition, astrocytoma and oligodendroglioma have similar morphological features and are commonly confounded diagnoses with large intraobserver variabilities^34,35^. Last, an expert intervention step is to screen out the non-representative prototypes further or re-assign to another category.

The purpose of the prototype approach is to measure the morphological similarities among all patches in each category for clustering and then select the representative patches from the major clusters. In this case, noisy samples or outliers will be excluded. The WSI-based method’s detail is as the following: first, all sampling patches in the same category are fed into the CNN model to derive deep features. Subsequently, the similarity matrix *S* is computed by pair-wise comparison of patches using cosine similarity, and each entity ${s}_{ij}$ in the matrix can be derived by Eq. (1)

1 $${s}_{ij}=\frac{G{\left({x}_{i}\right)}^{T}G({x}_{j})}{{\Vert G({x}_{i})\Vert }_{2}{\Vert G({x}_{j})\Vert }_{2}},$$

where $G({x}_{i})$ or $G({x}_{j})$ is the deep feature vector derived from the forward propagation of convolutional layers of the *i*th or *j*th sampling patch (${x}_{i}$ or ${x}_{j}$) in the CNN model. The entity ${s}_{ij}$ reflects the similarity between two arbitrary sampling patches. A similarity threshold, *s*_*t*_, is set to be the average value of all pair-wise similarity values in a category. The measurement of density for the *i*th sampling patch, ${\rho }_{i}$, is computed by Eq. (2).

It counts how many patches over the total *m* patches in a category.

2 $${\rho }_{i} = \sum_{j=1}^{m}sign\left({s}_{ij}-{s}_{t}\right).$$

For prototype selection in each category, the largest similarity value indicates the most representative index of the category. An ideal prototype set is able to distinguish all categories. To meet the requirements, another index of  is to measure the diversity of prototypes. The diversity index for each patch in a category is designed by Eq. (3)^36^.3.

**Figure 4:**
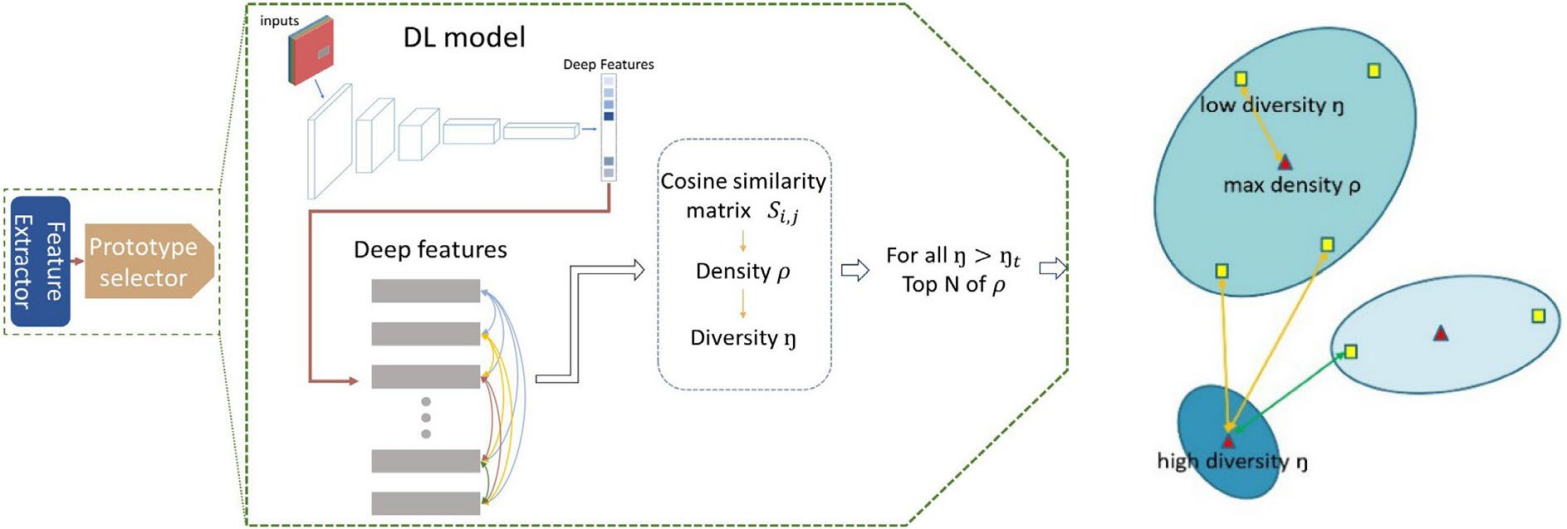
Prototype selection in each category. Left: The pipeline of prototype selection. Right: Measurements of density and diversity.

Figure 4 shows the scheme of prototype selection for each category with the rules as follows: All sampling patches for a category are fed into the CNN model for feature extraction. Subsequently, the similarity matrix *S* is derived by performing pair-wise similarity comparison among patches in the category. Afterwards, the measurements of density (${\rho }_{i}$) and diversity () of each patch are computed to determine the selection priority and selection condition. All patches in the category are ranked based on their corresponding density value $\rho$, which reflects the selection priority. In addition, the diversity threshold  is set to avoid the selected prototypes being too similar, resulting in redundancy during selection.

In Eq. (3), the sampling patch with the highest density value, i.e., ${\rho }_{max}$, among all sampling patches in a category will be assigned the top selecting priority, and it will also be assigned a large diversity index to surpasses the diversity threshold . For other sampling patches, the diversity index  measures the feature distance to the most similar patch with a higher density value $\rho$.

Accordingly, if the *i*th sampling patch is the prototype candidate, we check the  to ensure it is greater than the diversity threshold to retain high diversity among the selected prototypes within a category.

**Figure 5:**
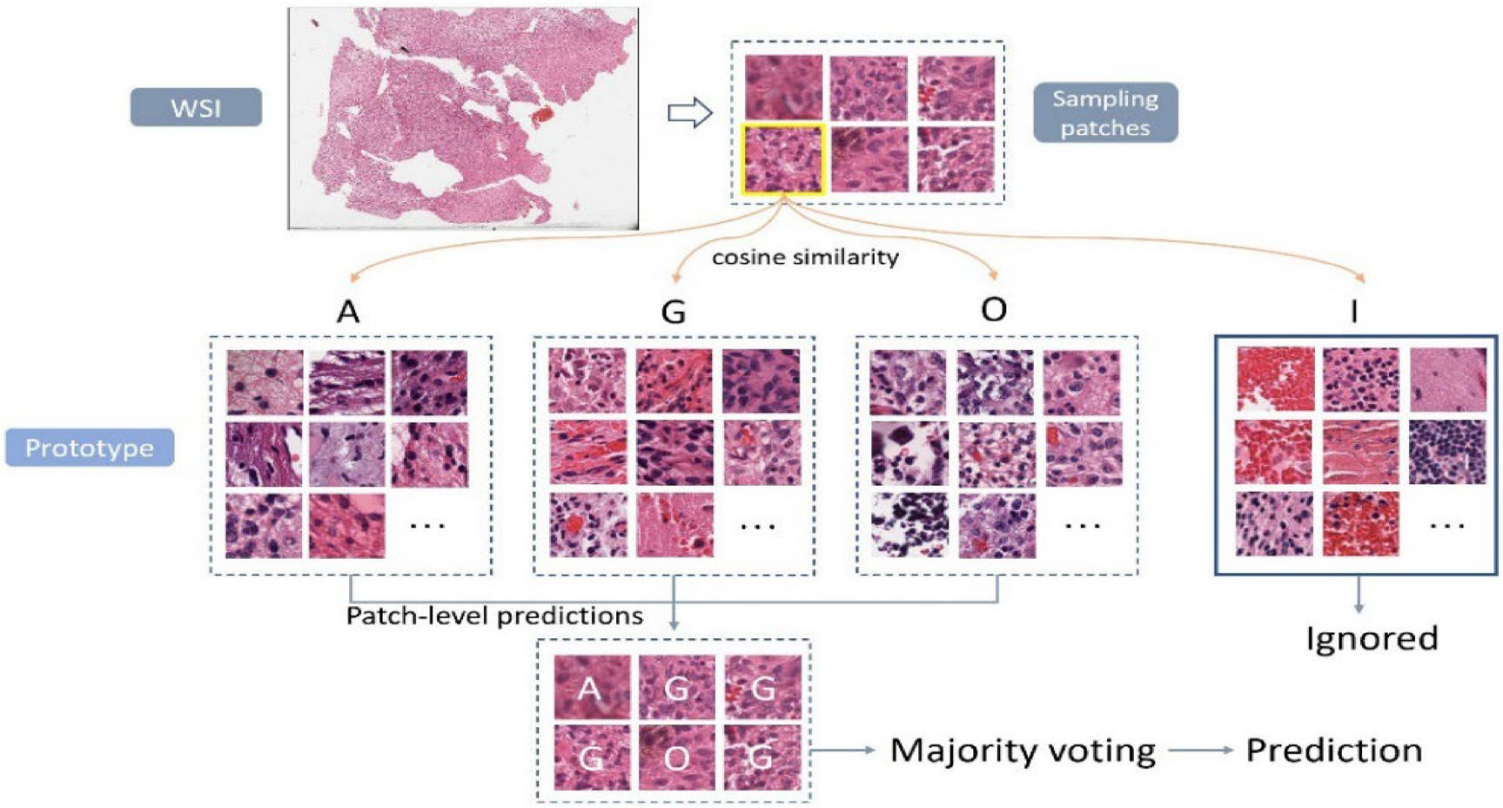
Example of case-level inference in the proposed WSI-approach.

In the inference phase, several patches are extracted from each WSI, and then fed into the CNN model for feature extraction. For each sampling patch, the cosine similarity is computed between the extracted feature and the deep features of each prototype in each category. Each patch is classified into the category with the highest average of cosine similarities among prototypes. Finally, a majority voting from all predictions of samples is performed to determine the final label prediction for the case. Figure 5 illustrates an example of case-level inference using the proposed WSI-based approach. Notably, we employ an additional category (I) for irrelevant classification of gliomas subtype. Sampling patches of the category (I) will be ignored in the voting process.

### MRI-based approach

**Figure 6:**
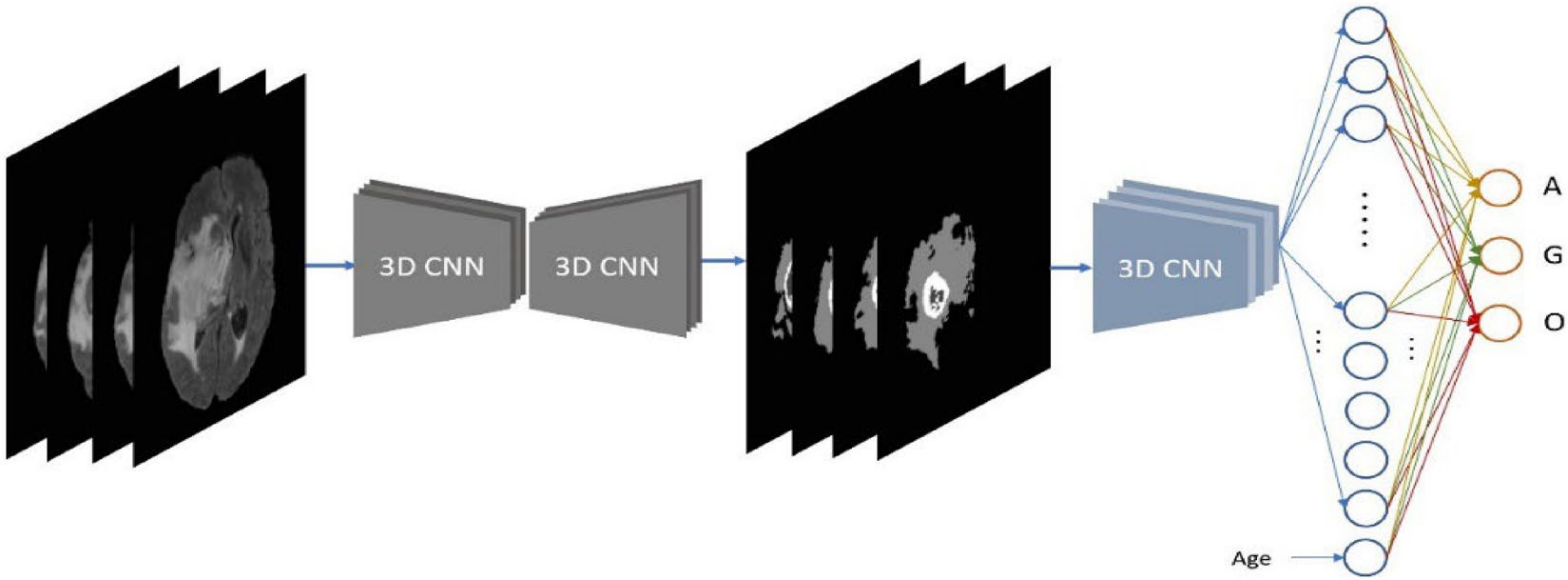
Pipeline of the proposed MRI-approach.

For the mpMRIs-based approach, we employ a cascade deep learning-based method. Brain tumors are firstly segmented using a 3D CNN model^37,38^, and then the segmentations are fed into another 3D CNN model for glioma subtype classification. The pipeline of the proposed MRI-based approach is shown in Fig. 6. Accurate segmentation of brain lesions leads to an outstanding performance on brain tumor classification. Since the intensity of MRI varies across all cases, intensity normalization is desirable to reduce the bias. In our experiments, a z-score normalization is applied for all MRIs. In doing so, all voxel values are subtracted by the mean and divided by the standard deviation of the brain region. In addition, several data augmentations are applied in the training phase of both segmentation and classification, such as rotation, random flipping, and affine translation. According to a report of 2008–2011, age is associated with brain tumor subtype^2^. Therefore, the age of the patient is also taken into consideration for glioma subtype classification.

### Label agreement

For the WSI-based approach, the prediction is derived by choosing the glioma subtype with the highest votes. In addition, the distributions of votes among all subtypes, regardless of the category (I), turn into probabilities indicating the confidence score for each glioma subtype. While for the MRI-based approach, the prediction probability for each class can be retrieved directly from the CNN classification network. According to the guideline of CNS glioma classification defined by WHO, histopathology information is the primary image source for the glioma subtype classification. Thus, we primarily consider the WSI-based result as the final prediction, but also take the mpMRIs-based result as a complementary reference when the confidence index of the WSI-based result is less than the pre-defined threshold, *C*_*t*_. In such a strategy, the final consensus of glioma subtype classification is decided by the fusion of WSI and mpMRIs information. We believe that integrating WSI and mpMRIs offers a more robust and reliable result than using a single image type.

## Datasets and experiments

### Ethics approval and guidelines

In this study, the MRI images and pathology images sourced from two pubic dataset: multimodal Brain Tumor Segmentation Challenge (BraTS) 2020^39–45^ and Computational Precision Medicine: Radiology-Pathology Challenge on brain tumor classification (CPM-RadPath) 2020^31^. Approval was granted on the grounds of existing datasets. Informed consent was obtained from all of the patients in this study. All methods were carried out in accordance with relevant guidelines and regulations. Ethical approval for the use of these data was obtained from the ethics committee of University of Pittsburgh.

### Dataset

The BraTS 2020 training data contains 369 cases including 76 low-glioma grade (LGG) patients and 293 high-glioma grade (HGG) patients. For each case, there are multiparametric MRIs (mpMRIs) and the corresponding ground truths of brain tumors. The mpMRIs include T1-weighted MRI (T1), T1-weighted MRI with contrast enhancement (T1ce), T2-weighted MRI (T2), and T2-weighted MRI with fluid-attenuated inversion recovery (T2-FLAIR). Each modality of each case has a size of $240\times 240\times 155$. The ground truth of a tumor segmentation contains multiple tumor subtypes, such as tumor tissues of peritumoral edema (ED), enhancing tumor (ET), and necrosis/non-enhancing tumor (NCR/NET).

For the data from CPM-RadPath 2020, the training dataset is comprised of 221 cases with paired radiology and digital pathology images. Within the 221 cases, there are 54, 34, and 133 cases for lower grade astrocytoma, *IDH-mutant* (A), oligodendroblioma, *1p/19q codeltion* (O), and glioblastoma and diffuse astrocytic glioma with molecular features of glioblastoma, *IDH-wildtype* (G), respectively. In addition, there are 35 and 73 cases for the validation and testing sets in the CPM-RadPath 2020 challenge, respectively. It notices that the challenge organizer privately owns the ground truth of the glioma subtype of the validation and testing data. In the validation phase, participants submit the prediction results to the challenge for online evaluating the algorithm. However, participants are only allowed to submit the algorithm wrapped in a Docker container in the testing phase, and the organizer executes the algorithm for final ranking.

### Evaluation metrics

Three metrics are utilized for performance evaluation in the challenge of CPM-RadPath 2020 for glioma subtype classification, which are micro-F1, Cohen’s Kappa Coefficient, and balanced accuracy. In the tasks of multi-class classification, the micro-F1 is equivalent to the overall accuracy, as Eq. (4).

4 $$Micro{\text{-}}F1=\frac{total\, TruePositives}{total \,\#\, cases} \left(for\, multi{\text{-}}class\right).$$

The Cohen’s Kappa Coefficient ($\kappa$) is to measure inter-rater and intra-rater reliability for categorical items, and the definition is as Eq. (5).

5 $$\kappa =\frac{{p}_{o}-{p}_{e}}{1-{p}_{e}},$$

where *p*_*o*_ is the relative observed agreement among raters, and *p*_*e*_ is the hypothetical probability of chance. Lastly, the balanced accuracy computes the average of the proportion corrects of each class individually, which is formulated in Eq. (6).

6 $$Balanced \,accuracy= \frac{\sum_{i=1}^{i=n}{Recall}_{i}}{n},$$

where *n* indicates the number of classes in the task.

### Glioma subtype classification

For the WSI-based approach, 300 patches are sampled from each case of WSI and are fed into the proposed pipeline for patch-level classification in our experiments. Subsequently, the glioma subtype for each case can be determined by choosing the subtype with the highest votes, and the vote distributions are normalized into the probabilities as the confidence scores for each glioma subtype.

**Figure 7:**
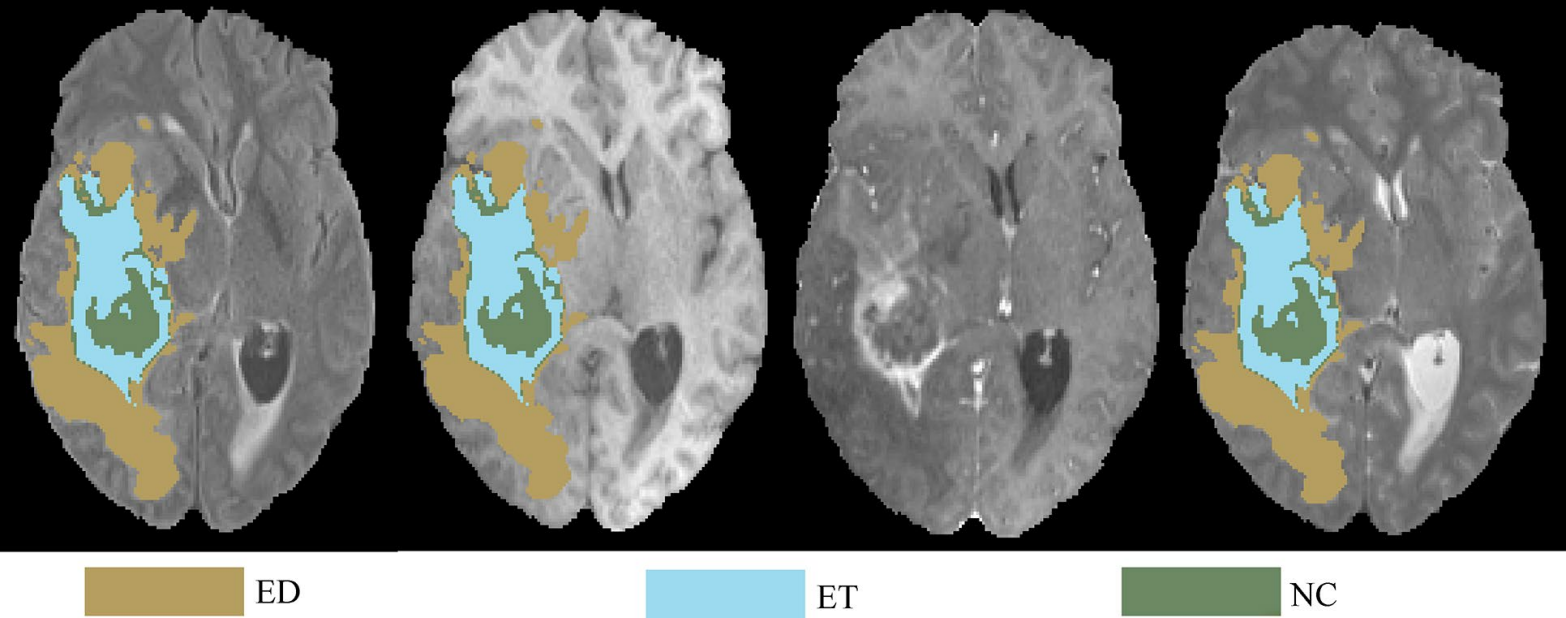
An example of segmentation results of three subregions of tumor, which are peritumoral edema (ED) in green, enhancing tumor (ET) in red, and necrosis or non-enhancing tumor (NCR/NET) in blue.

On the other hand, for the MRI-based approach, a 3D CNN model of ResUNet^37,38^ is trained on the dataset from the challenge of BraTS 2020 to perform tumor subregion segmentation. The task targets three tumor subregions, including peritumoral edema (ED), enhancing tumor (ET), and necrosis or non-enhancing tumor (NCR/NET). The segmentation results of three targeted tumor subregions are shown in Fig. 7. Subsequently, the segmentation model is applied on all the MRI cases in the training dataset from CPM-RadPath 2020, and the segmentation results are fed into another 3D CNN model of ResNet^31,33^ to train the relations between tumor morphology and glioma subtypes.

It notes that the CPM-RadPath 2020 has identical datasets as CPM-RadPath 2019. Since the glioma subtype of ground truth for each case in the testing set from the challenge of CPM-RadPath 2020 is not publicly available, in our experiments, the performance on the validation set from the challenge is focused for evaluation and comparison.

## Result

The performance of the two approaches and the scheme of label agreement is listed in Table 1. The confidence threshold in the scheme of label agreement, *C*_*t*_, is empirically set to be 0.6 in our experiment. The comparison shows a promising result that a label fusion-based predictions using the proposed method outperform the ones from single approach.

**Table 1:** Performance of two approaches and the scheme of label agreement.

Phase	Method	Case	F1-score	Kappa	Balance_acc	Average
Validation	MRI-based	35	0.771	0.627	0.698	0.699
	WSI-based	35	0.886	0.798	0.777	0.821
	Fusion	35	0.886	0.801	0.8	0.829
Test	Fusion	73	0.712	0.505	0.654	NA

In addition, we also compare our result to the other top-ranked teams in the validation phase, as shown in Table 2. The results show that our proposed method achieves a competitive performance among all on the validation set. Moreover, we participate the CPM-RadPath testing phase. It notices that all participants are required to submit the algorithm wrapped with Docker in the testing phase of CPM-RadPath 2020. The challenge organer excutes the algorithm, and ranks the performance. Our result ranks the third place in the testing phase. It indicates that the proposed method offers a competitive performance on glioma subtype classification.

**Table 2:** Performance comparison among state-of-the-art approaches in CPM-RadPath 2019/2020.

Phase	Method	F1-score	Kappa	Balance_acc	Average	Ranking
Valid	Ma et al.^27^	0.943	0.903	0.833	NA	NA
	Pei et al.^26^	0.829	0.715	0.749	0.764	NA
	Chan et al.^29^	0.72	NA	NA	NA	NA
	Xue et al.^28^	0.849	NA	NA	NA	NA
	Our method	0.886	0.801	0.8	0.829	NA
Test	Pei et al.^26^	0.603	0.39	0.596	NA	2nd (2019)
	Our method	0.712	0.505	0.654	0.654	3rd (2020)

All references are coming from the top teams in the CPM-RadPath 2019/2020.

## Discussion

**Figure 8:**
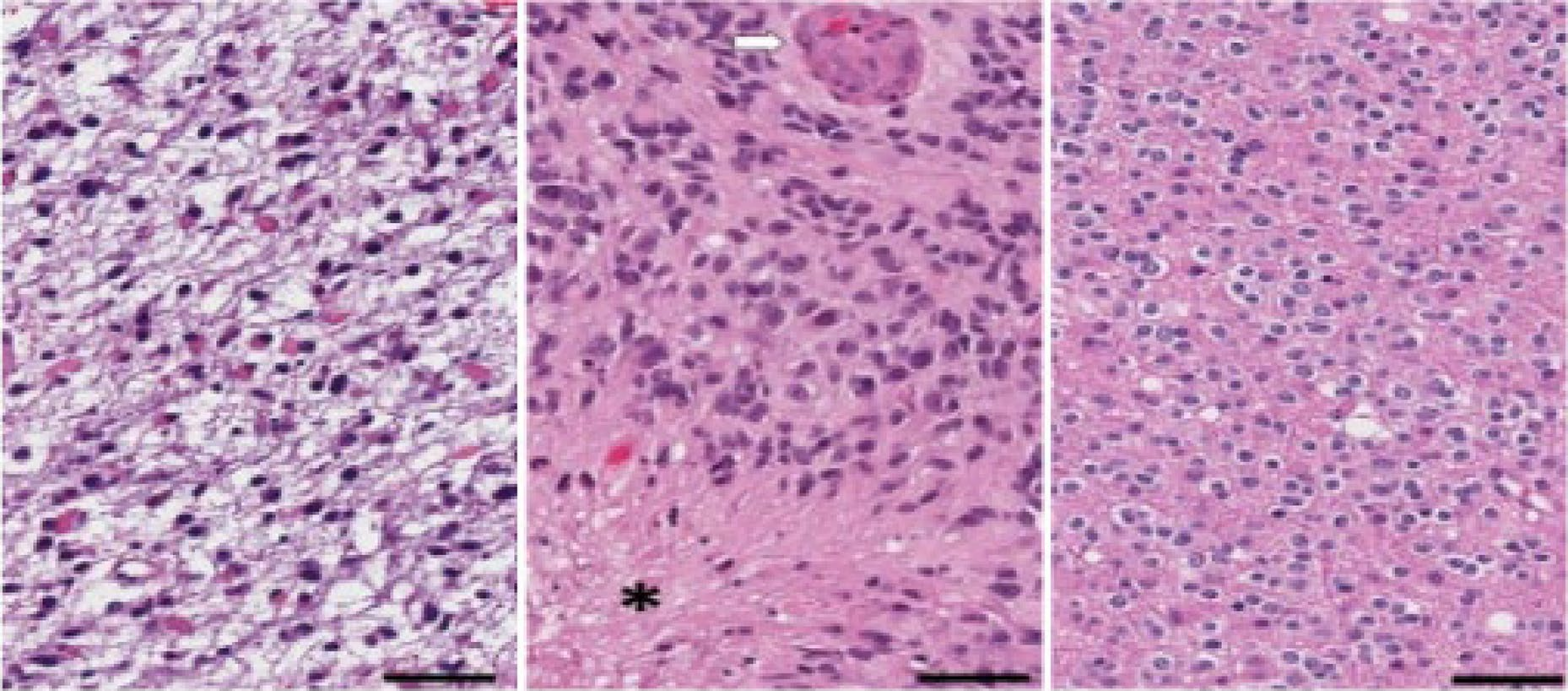
Pathological morphologies corresponding to each subtype of glioma. The scale bar at the bottom-right cornor indicates the actual size of 50 µm at the magnification. Left: astrocytoma (A). Middle: glioblastoma (G). Right: oligodendroglioma (O).

From the pathological perspective, the morphologies of glioma subtypes are distinct. As it can be observed in Fig. 8-Left, astrocytoma revealed hypercellularity with irregular and hyperchromatic nuclei in the fibrillary background. The pink and abundant cytoplasmic with eccentric nuclei (gemistocytic differentiation) occasionally showed in this group. While glioblastoma, as shown in Fig. 8-Middle, is derived from astrocytoma; hence, many morphologic findings are shared. However, few discriminatively histopathologic features can distinguish them, such as glomerular endothelial proliferation (pointed by a white arrow) and tumor necrosis (the area marked with a star-shaped sign) only show in glioblastoma. Last, the oligodendroglioma appears round shape nuclei with open chromatin and artifactual cytoplasmic retraction, leading to the “fried egg” appearance, as shown in Fig. 8-Right.

**Figure 9:**
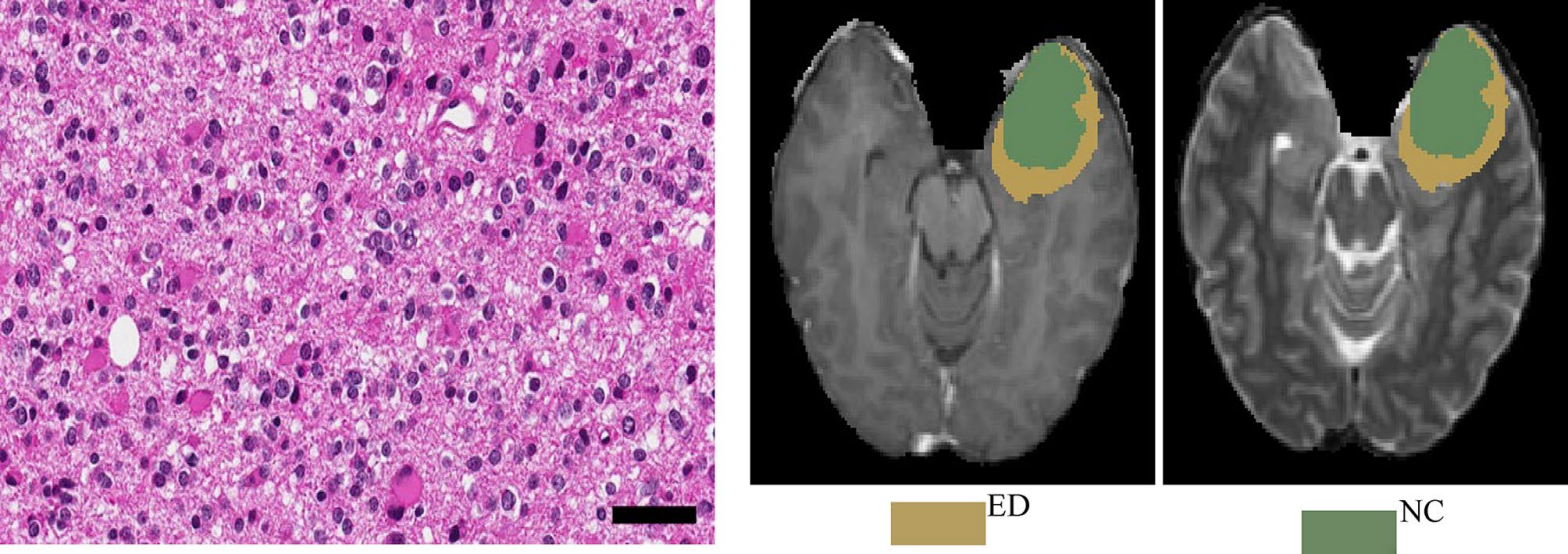
One of the cases that was overdiagnosed by the WSI-based approach but successfully corrected by the MRI-based approach after the proposed label agreement scheme. Left: Pathological observation. Right: The brain tumor segmentation on MRI.

Figure 9 shows an example that the prediction from the MRI-based approach successfully corrected the final diagnostic result using the proposed label agreement scheme. In this case, the prediction from the WSI-based approach is overdiagnosed astrocytoma as glioblastoma, which might occur because both of them have shared many morphologic features. The weight of decisive features, including necrosis or vascular proliferation, should be recruited more for model training to increase the chance of separation. Fortunately, this misinterpretation is corrected by the mpMRIs-based approach. MRI is a powerful source to detect necrosis and vascular abnormality by evaluating the amount of enhancement, degree of heterogeneity, and liquid components. As it can be observed in Fig. 9, there is no enhancement or necrosis identified in MRI. It can be a clue for the computer-aided system to classify the case to the category of astrocytoma, instead of glioblastoma. The voting distribution of the case by the WSI-based approach and the probabilities of subtype predictions by the mpMRI-based approach are shown in Table 3. The assigned labels of WSI-based and mpMRI-based method are “G” and “A”, respectively. However, since the confidence index (0.5069) by the WSI-based method is less than the threshold value (0.6), the final label agreement assigns “A” for the correction.

**Table 3:** Voting distribution and predicting probabilities by using WSI-based and mpMRI-based methods, respectively.

Method	Astrocytoma	GBM	Oligodendroglioma	Assigned label
WSI-based	0.3844	**0.5069**	0.1088	G
mpMRI-based	**0.9413**	0.0145	0.0441	A
Final agreement	–	–	–	A

The maximum probability is in bold.

**Figure 10:**
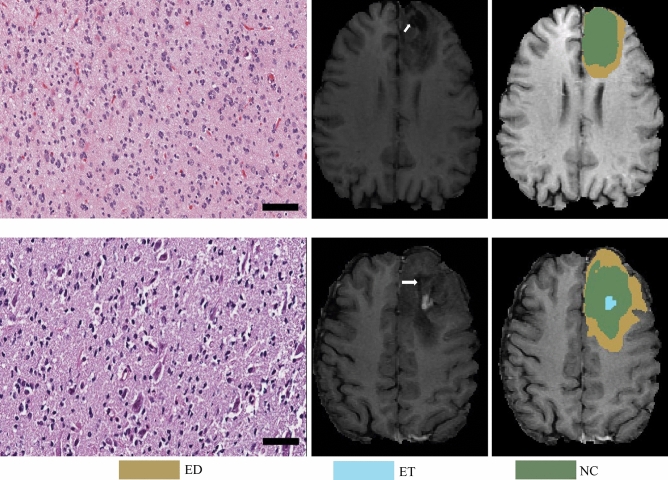
Two cases of misinterpretation from Oligodendroglioma to Astrocytoma. From left to right: WSI, corresponding T1ce and its segmentation. (Top): Case of CPM20_TCIA10_239_1, (Bottom): Case of CPM20_TCIA1_387_1. The arrow on T1ce points to cavity.

However, misclassification also occurs. In the experiment, there are another two cases misinterpreted, as shown in Fig. 10. For the first case, in Fig. 10-(Top), it is mis-classified oligodendrndroglioma as an astrocytoma by the WSI-based approach. There are two explanations for the misinterpretation: First, this case belongs to a higher grade oligodendroglioma (WHO grade III) and shows a more severe degree of nuclear atypia, mimicking astrocytoma. Second, the slide is mainly located in the infiltrating part mixing with tumor and adjacent brain pathologic images. Unfortunately, the mpMRIs-based approach does not correct the misclassification by following the strategy of label agreement. The prediction of the WSI-based approach is Astrocytoma with confidence of 0.93, while the prediction of the mpMRI-based approach is GBM. However, the ground truth label of this case is Oligodendroglioma. Figure 10-(Bottom) shows another case of misinterpretation. It is misdetermined oligodendroglioma to astrocytoma by the WSI-based approach due to poor fixation and staining procedure. In addition, the tumor cells have revealed marked pyknosis and dark nuclei without nuclear details, leading to misinterpretation. Though, some features of oligodendroglioma can be identified, such as artifactual cytoplasmic retraction. It may be a dilemma for the model to make decisions with such two contradictory features observed. The prediction of the mpMRI-based approach is same as that of the WSI-based approach, but both are misclassified. Unfortunately, the ground truths of brain tumor segmentation are not available for public. We also notice that both cases contain a cavity after post-surgery, which may result in the misclassification.

Though the proposed method produces a competitive result, there are some limitations. First, we use both WSI and mpMRIs for the classification. To achieve the best result, we assume that an accurate brain tumor subregion results in a good classification. In doing so, we use an extra dataset from BraTS 2020, which limits its application. Second, the proposed method is sensitive to some empirical parameters, such as the threshold of confidence index, the number of extracting patches, etc. Third, the relatively small size of experimental data is a drawback, which widely exists in deep learning-based methods. Forth, the proposed method requires a qualified professional or expert intervention to screen out the non-representative prototypes.

## Conclusion

In this study, we propose a novel hybrid fully convolutional neural network (CNN)-based method for glioma subtype classification using both whole slide image (WSI) and multiparametric magnetic resonance images (mpMRIs). It is comprised of two methods: a WSI-based method and a mpMRIs-based method. For the WSI-based method, we categorize the glioma subtype using a 2D CNN on WSIs. For the mpMRI-based method, we also develop a 3D CNN-based method by analyzing the mpMRI. The mpMRIs-based method consists of brain tumor segmentation and classification. We classify the glioma subtype primarily on WSI-based results with the guidance of the mpMRIs-based prediction when the confidence index of the WSI-based result is less than the pre-defined threshold. The experimental results show that the final label fusion-based predictions achieve a superior result and offer a competitive performance.

## Data Availability

In this study, Our data are coming from both https://www.med.upenn.edu/cbica/brats2020/data.html and https://www.med.upenn.edu/cbica/cpm2020.html. The data can be found: https://www.med.upenn.edu/cbica/brats2020/data.html (BraTS 2020), and https://miccai.westus2.cloudapp.azure.com (CPM-RadPath 2020).
